# Editorial: Exercise and chronic disease

**DOI:** 10.3389/fpubh.2024.1375156

**Published:** 2024-02-12

**Authors:** Yanan Zhao

**Affiliations:** Department of Sports Science and Physical Education, Nanjing Normal University, Nanjing, China

**Keywords:** exercise, chronic disease, physical activity, behavior, quality of life

With the development of medical science and technology, human longevity has been extended and advanced medical treatments come to be true. Human beings are happy to welcome the prolonged lifespan, while the bad news is the extended duration of bedtime, which requires special care in daily life and in sick time because of illness or chronic disease. The fast pace of life and the vast pressure of work not only harm the health status of young, middle-aged and older adults but also deteriorate the life quality of adolescents and children. Of all the health-related risk factors, a deficiency in daily physical activity is always the crucial element of degraded life capacity (1). In other words, there are significant, solid and positive associations between regular exercise behaviors and the health level of human beings. It has been well-evidenced that exercise can prevent various diseases and improve physical and mental health (2). As a long-lasting condition that can be controlled but not cured, chronic disease is no longer taken as a single problematic dimension but the result of multiple body systems acting together and complicated mechanisms interacting with each other. Besides, the coverage of chronic concepts has extended from heart disease, diabetes and cancer to mental problems such as depression and anxiety. Traditional treatments may not fit the preventative purpose and regular exercise behavior as the endogenous medicine cabinet can provide sufficient self-protection to maintain sound health in daily life (3).

Under the topic of exercise and chronic disease, the included papers cover diverse aspects of exercise to chronic-related research. Topics range from the acute effects of various exercise forms on executive function and cerebral hemodynamics in hospitalized Type 2 Diabetes Mellitus (T2DM) patients (Wang H. et al.) to investigating the associations between grip strength, comorbidities, and all-cause mortality in older hypertensive adults (Wang Y. et al.). Cohort studies explore the relationships between cardiorespiratory fitness, body mass index, cardiovascular disease, and mortality in young men (Gorny et al.). Additionally, a systematic review and meta-analyses focused on the effects of aquatic exercises on postmenopausal women's physical fitness and quality of life (Zhou et al.). With the background of COVID-19, the impact of pre-pandemic physical activity on COVID-19 infection and mortality is explored, drawing evidence from the National Health Insurance Service (Park et al.). Other studies investigate ideal cardiovascular health in rural northeast China (Shao et al.), the joint association of physical activity and sedentary behavior with metabolic syndrome in urban men aged 60+ (Lou et al.), and trends in the rate of regular exercise among adults in Jiangsu, China, from 2010 to 2018 (Su et al.). The importance of careful consideration of overall movement behaviors for preventing, treating, and following up on cancer risks and patients is also emphasized (Ennequin et al.). In summary, the focused topic provides new evidence on exercise improving health and contributes to deepening the understanding of exercise benefits to human beings.

By dialectical thinking, exercise can prevent chronic diseases of human beings, and people with chronic diseases would enhance or give up exercise behaviors depending on the symptoms of chronic disease. Scientific researchers have plunged into exploring the dosage of exercise prescription for various chronic diseases, while a huge gap still exists between research and practice. It is necessary to re-think the essentials of exercise itself. Fundamentally speaking, exercise is a kind of human behavior. Since ancient times, physical activity has been one of the essential behaviors for sustaining human existence in the world. In modern society, exercise serves as the cornerstone for maintaining the physiological functions of the human body. With the advanced industrial and technological developments, people are increasingly inclined toward a sedentary lifestyle, which contradicts the basic needs of human life. Along with further elucidation of physiological mechanisms underlying exercise promoting health, there is a pressing need to focus on the strategies to sustain and enhance daily physical activity and exercise-the basic behaviors of human beings.

The following suggestions are therefore provided for future research and clinical practice. At the individual level, it is crucial to raise awareness among the general public about the benefits of exercise and scientific training methods and provide opportunities for individuals to engage in exercise and diversify their choices. From the perspectives of family, school, and community, future research should consider the unique characteristics of different regions or countries, creating a sport-friendly environment with facilities and equipment suitable for diverse populations to exercise. Additionally, it should be highly recommended to integrate exercise as a necessary part of lifestyle with small-scale events weekly and large-scale competitions monthly. At the national level, it is imperative to incorporate exercise into social and cultural development by promoting, encouraging, and supporting individuals' engagement in exercise.

## Author contributions

YZ: Writing—original draft, Writing—review & editing.
